# Development of a Hybrid Biomimetic Enamel-Biocomposite Interface and a Study of Its Molecular Features Using Synchrotron Submicron ATR-FTIR Microspectroscopy and Multivariate Analysis Techniques

**DOI:** 10.3390/ijms231911699

**Published:** 2022-10-02

**Authors:** Pavel Seredin, Dmitry Goloshchapov, Vladimir Kashkarov, Yury Khydyakov, Dmitry Nesterov, Ivan Ippolitov, Yuri Ippolitov, Jitraporn Vongsvivut

**Affiliations:** 1 Solid State Physics and Nanostructures Department, Voronezh State University, University sq.1, 394018 Voronezh, Russia; 2 Department of Pediatric Dentistry with Orthodontia, Voronezh State Medical University, Studentcheskaya st. 11, 394006 Voronezh, Russia; 3 Australian Synchrotron (Synchrotron Light Source Australia Pty Ltd.), 800 Blackburn Rd, Clayton, VIC 3168, Australia

**Keywords:** biomimetic, bioinspired materials, hybrid layer, nanocrystalline carbonate-substituted hydroxyapatite, polar amino acids, synchrotron infrared microspectroscopy

## Abstract

Using a biomimetic strategy and bioinspired materials, our work proposed a new technological approach to create a hybrid transitional layer between enamel and dental biocomposite. For this purpose, an amino acid booster conditioner based on a set of polar amino acids (lysine, arginine, hyaluronic acid), calcium alkali, and a modified adhesive based on BisGMA and nanocrystalline carbonate-substituted hydroxyapatite are used during dental enamel restoration. The molecular properties of the hybrid interface formed using the proposed strategy were understood using methods of multivariate statistical analysis of spectral information collected using the technique of synchrotron infrared microspectroscopy. The results obtained indicate the possibility of forming a bonding that mimics the properties of natural tissue with controlled molecular properties in the hybrid layer. The diffusion of the amino acid booster conditioner component, the calcium alkali, and the modified adhesive with nanocrystalline carbonate-substituted hydroxyapatite in the hybrid interface region creates a structure that should stabilize the reconstituted crystalline enamel layer. The developed technology can form the basis for an individualized, personalized approach to dental enamel restorations.

## 1. Introduction

Human tooth enamel is the hardest tissue in the body and has excellent mechanical properties due to its unique biocomposite structural organization [1,2]. A variety of materials, such as composite resins, polyacid modified composites, glass ionomer cements, and various bioceramics have been developed to restore dental enamel [3,4]. However, a sustainable restoration has never been achieved due to the imperfect combination between these materials and natural enamel. Therefore, the durability of restorations is still an open question [5]. For this reason, the main strategy of modern restorative dentistry is the minimally invasive treatment with maximum preservation of the natural healthy tooth tissue [6]. In this case, the quality and durability of the restoration will be determined by the stability of the enamel to composite bond [7] as well as the state of the interface after the direct restoration procedure, which in turn, depends on the restoration techniques and the affinity of the materials used and the natural enamel [3,8].

Human tooth enamel is formed by a dense packing of highly mineralized hydroxyapatite cores conjugated with a protein matrix, but it is also permeable to certain ions and molecules [1,2,9]. Due to its specific chemical nature, enamel is susceptible to dissolution in various acids [10]. This property is widely used in dentistry for enamel restoration [11]. Acid etching, and in particular phosphoric acid, is still the optimal approach for restorations [3]. Moreover, for the integration of synthetic materials with enamel self-adhesive systems, tiller and total etch techniques are widely used. The conjugation of dissimilar materials is achieved by diffusion and adhesion of the adhesive monomers to the array of micropores formed by etching [7,12]. For all that, the convenience and ease of use of bonding systems does not take into account the individual characteristics of the patient, which can lead to insufficient adhesion to the enamel [5,13].

However, the durability and quality of the restoration can be significantly improved by the use of biocomposites and systems capable of chemically binding to calcium phosphates and promoting the biomineralization of distended enamel [14]. The scientific basis for this approach is the concept of the biomimetic restoration of human dental tissue. Similar to natural biomineralization, the strategy allows hierarchical structures to be obtained—biomimetic composites that are similar to natural dental tissue—due to the coordinated accumulation of bioinspired inorganic and organic components [15,16]. This technological approach combines interdisciplinary fundamental and applied tasks of creation, research, and the application of biomaterials in dental practice.

In terms of restorative dentistry, the strategy of biomimetic tissue engineering requires the involvement of amino acids (proteins) and minerals for tissue regeneration [17,18]. Amino acids used in the framework of the biomimetic approach can be adsorbed on the surface of natural dental tissue due to the formation of amide or carboxyl bonds and contribute to the formation of the required morphology [19,20]. Thus, the simultaneous co-precipitation of amino acids with various phosphate complexes from model (buffer) solutions is very attractive, which leads to the formation of hierarchical structures of different ordering [21,22,23].

It has been shown that the required morphological features of the regenerated tissue can be recreated by choosing specific amino acids and conditions of biomaterial formation [24]. Current breakthrough studies demonstrate that the use of buffer systems when forming a hybrid biomimetic layer, including amino acids and alkalis, should contribute to the improved integration of synthetic systems with the dental hard tissue [25,26,27,28]. The introduction of amino acids and alkalinization under given conditions should increase the hierarchical organization of the pretreated organomineral matrix of apatite [8,29,30,31]. Thus, in our previous work, it was shown that the pretreatment of enamel in the alkaline solution of Ca(OH)_2_ and an amino acid booster, and the following mineralization performed with the use of hydroxyapatite (HAp), resulted in the formation of a mineralized layer with homogeneous micromorphology and presumable orientation of HAp nanocrystals [32,33]. The binding of hydroxyapatite nanocrystals with an amino acid complex resulted in a considerable increase (~15%) in the nanohardness value in the mineralized layer as compared with a similar value of the intact natural enamel. Thus, a current and relevant question is the establishment of the mechanisms of interaction of the structural components (organic and inorganic) of biomimetic composites with natural enamel apatite [15,34]. This question demands careful all-round study.

Molecular spectroscopy techniques, which allow for the analyzing of biological samples without irreversible external influence, may be the most convenient here [35,36]. These techniques are well established and widely used to study various organomineral composites and hybrid nanofilled biomaterials [37,38]. Sensitivity to changes in the conformational environment of organic molecules allows the use of molecular spectroscopy methods to establish the formation of chemical bonds, as well as to determine the type of interaction in multicomponent biosystems of complex composition [39,40,41,42]. The coupling of spectroscopic methods with optical microscopy techniques and the use of synchrotron radiation sources makes it possible to study local submicron regions in samples of biological nature, such as bionanocomposites, as well as to determine the interaction mechanisms of individual components in their composition [43,44,45].

Thus, the goal of our work was to develop new approaches to human tooth enamel restoration within the framework of the biomimetic concept, as well as to study the molecular features of the formed hybrid biomimetic interface using synchrotron microspectroscopy techniques.

## 2. Materials and Methods

### 2.1. Sample Preparation Methodology

#### 2.1.1. Dental Tissue Samples

In our study, intact third tooth molars were used to develop new technological approaches to enamel restoration within the framework of the biomimetic concept. The teeth were extracted for orthodontic indications in patients (male and female) aged 18–25 years at the dental clinic of the Burdenko Voronezh State Medical University. The dental donors were physically healthy, had no bad habits, and did not smoke, which was confirmed by the individual outpatient records of the patients.

Tooth molars were extracted in accordance with relevant guidelines and regulations and data collection and handling followed the Helsinki declaration.

All donors provided their written consent for participation. The Ethics Committee of Voronezh State University affirmed the performed examination (number of permission 003.017-2019).

After extraction, the teeth were placed in separate vials containing 0.9% saline and 0.002% sodium azide and stored at 4 °C.

#### 2.1.2. Treatment of Dental Tissue

Initially, the occlusal upper part of the crown of the teeth was mechanically cleaned with a stiff brush. The teeth were then rinsed with a stream of distilled water and dried with a stream of air from an oil-free compressor.

Using an Er:YAG pulsed laser (2940 nm, duration 75–500 μs, frequency 10–50 Hz, PMax = 8 W) a Dental laser PLUSER (Lambda S.p.A., Brendola, Italy), a cylindrical cavity of ~2 mm depth and ~3 mm diameter was formed in the area of the chewing surface of each sample tooth enamel. The resulting cavity was washed with distilled water and dried with air flow from a compressor. To form a characteristic enamel morphology (array of microporosities), to provide a micromechanical interaction with the adhesive, as well as in accordance with the instructions of the manufacturer of the commercial bonding system, the cavity walls in the enamel were selectively treated with etching gel based on 37% phosphoric acid (Etching gel, KORMED—R, Russia) for 30 s. The cavity walls and bottom were then rinsed with distilled water and dried with an air stream from an oil-free compressor.

The 20 samples prepared in this way were randomly divided into four equal groups to test different approaches to forming the enamel-composite interface.

#### 2.1.3. Forming Interfaces

To create samples (interfaces) of the first type—S_I_, we used a bonding system including a conditioner, bioprimer, and a commercial universal adhesive based on BisGMA (Polysciences, Warrington, PA, USA, code 03344) [25,46] and a dental compomer material DyractXP (Dentsply Sirona CIS, Bensheim, Germany) [47]. At the beginning of the procedure, the walls and the bottom of the cavity formed in the enamel were treated with conditioner (Vladmiva-Pharma Dental, Belgorod, Russia) for 30 s. Then, using microbrashers, the cavity was treated with 0.1 mL of bioprimer for 20 s.

The bioprimer contains compomer components (ethylene glycol methyl ester 30–85%, urethane dimethacrylate 1–15%, diglycidyl methacrylate hydrophilic monomer 1–15%) and a complex of polar amino acids (histidine 0.01–0.2%, lysine 0.05–0.4%, arginine 0.2–1.6% of the total primer weight), which support the synthesis of basic proteins. Bioprimer is used to introduce the bonding components into the dentinal tubules and form a hybrid layer with the prepared dentine tissue.

After treating the cavity, bioprimer was distributed on the surface of the cavity using air flow from the oil-free compressor for 5 s. After exposure of the prepared cavity for 20 s, a flowable universal adhesive on the basis of BisGMA was applied and distributed on the surface of the cavity via the air stream from the oil-free compressor for 5 s. Then, the adhesive was photopolymerized for 5 s using a light curing unite LED B Cordless (Woodpecker, Beijing, China), light wave: 420 nm to 480 nm, light intensity: 100 mW/cm, power 1000 mW/cm^2^–1700 mW/cm^2^. Finally, DyractXP light-curing composite was applied and photopolymerized following the manufacturer’s instructions.

To create specimens (interfaces) of the second type—S_II_, a conditioner, bioprimer, calcium alkali, BisGMA-based adhesive, and DyractXP dental compomer material were used.

The cavity formed in the enamel using microbrushes was treated once with calcium hydroxide for 30 s to obtain an alkaline environment (pH > 11). The cavity was then rinsed with distilled water and dried with oil-free compressor air flow for 5 s. After that, the cavity was treated with conditioner for 30 s. Then, using microbrashers, the walls and the bottom of the cavity formed in the enamel were treated with bioprimer in a volume of 0.1 mL for 20 s. After treating the cavity, the bioprimer was distributed on the surface of the cavity via air flow from the oil-free compressor for 5 s. After exposure of the prepared cavity for 20 s, a flowable universal adhesive on the basis of BisGMA was applied and distributed on the surface of the cavity by the air stream from the oil-free compressor for 5 s. Then, the adhesive was photopolymerized for 5 s. Finally, DyractXP light-curing composite was applied and photopolymerized following the manufacturer’s instructions.

To create samples (interfaces) of the third type—S_III_, the following were used: amino acid booster, bioprimer, calcium alkali, modified adhesive based on BisGMA and the DyractXP dental compomer material.

The cavity formed in the enamel was treated once for 30 s with calcium hydroxide using microbrushes to obtain an alkaline environment (pH > 11). The cavity was then rinsed with distilled water and blown with oil-free compressor air flow for 5 s and then treated with an amino acid booster for 30 sec. Using microbrashers, the walls and bottom of the cavity formed in the enamel were treated for 20 s with 0.1 mL of bioprimer. After treating the cavity, the bioprimer was distributed on the surface of the cavity by air flow from the oil-free compressor for 5 s. After exposure of the prepared cavity for 20 s, a flowable modified adhesive based on BisGMA was applied and distributed on the surface of the cavity by air flow from the oil-free compressor for 5 s. Then, the adhesive was photopolymerized for 5 s. Finally, the DyractXP light-curing composite was applied and photopolymerized following the manufacturer’s instructions.

An amino acid booster, bioprimer, calcium alkali, a modified BisGMA-based adhesive, and the DyractXP dental compomer material were used to create samples (interfaces) of the fourth type—S_IV_.

The cavity formed in the enamel was treated three times with calcium hydroxide using microbrushes to obtain an alkaline environment (pH > 11). After each treatment, the cavity was rinsed with distilled water and blown through with oil-free compressor air flow for 5 s. Between the calcium hydroxide treatments, the cavity was treated with an amino acid booster for 30 s. Thus, the total exposure time with calcium hydroxide was 3 × 30 = 90 s, and with amino acid booster 2 × 30 = 60 s. This procedure was performed to: completely neutralize the acidic environment formed after the action of orthophosphoric acid; consolidate the effect of formation in the near-surface layers of the enamel alkaline environment (pH > 11); to activate the formation of the molecular bonds of hydroxyapatite–amino acid throughout the cavity surface.

Then, using microbrashers, the walls and the bottom of the cavity formed in the enamel were treated for 20 s with 0.1 mL of bioprimer. After treating the cavity, the bioprimer was distributed on the surface of the cavity by air flow from the oil-free compressor for 5 s. After exposure of the prepared cavity for 20 s, a flowable modified adhesive based on BisGMA was applied and distributed on the surface of the cavity by air flow from the oil-free compressor for 5 s. Then, the adhesive was photopolymerized for 5 s. Finally, the DyractXP light-curing composite was applied and photopolymerized following the manufacturer’s instructions.

#### 2.1.4. Experiment Design

The design for creating interfaces is described schematically in Table 1.

#### 2.1.5. Sectioning

The prepared samples were divided into plane-parallel segments similar to those investigated in [48,49]. For this purpose, we used a low-speed water-cooled diamond saw (IsoMet 1000, Buehler, UK). The cutting wheel rotation speed was 100-rpm. The obtained slices of hard tissue containing the restoration areas were subjected to gentle grinding and polishing with a diamond abrasive.

### 2.2. Materials

All chemical components were purchased from Sigma-Aldrich (St. Louis, MO, USA).

#### 2.2.1. Conditioner

This consists of a complex of low-concentration (up to 12%) saturated and unsaturated polyfunctional organic acids (maleic acid—6%, polyacrylic acid—5%, citric acid—7%, distilled water). To prepare the amino acid solution, the raw components were dissolved in the ultra-pure water (provided with Millipore Milli-Q gradient ultrapure water system) and this mixture was subjected to ultrasound stirring (Q55 Sonica 55 W) with an amplitude of 50% for 5 min.

#### 2.2.2. Modified Conditioner with Amino Acids Booster

The main polar amino acids (arginine: 72%, lysine: 18%, histidine: 9%) were added to the composition of the original conditioner. The use of an amino acid booster should promote the formation of hierarchical structures based on phosphate complexes and amino acids of different ordering. Amino acids were mixed with the original conditioner using an ultrasound homogenizer, QSonica 55 W (QSonica LLC, Newtown, CT, USA).

#### 2.2.3. Bioprimer

This contains compomer components (ethylene glycol methyl ester 30–85%, urethane dimethacrylate 1–15%, diglycidyl methacrylate hydrophilic monomer 1–15%) and a complex of polar amino acids (histidine 0.01–0.2%, lysine 0.05–0.4%, arginine 0.2–1.6% of the total primer weight), which support the synthesis of basic proteins. Bioprimer was used to introduce bonding components into the dentinal tubules and to form a hybrid layer with the prepared dentine tissue.

#### 2.2.4. Calcium Hydroxide

Pasty calcium hydroxide was obtained by mixing a commercial material Trioxident (Vladmiva-Pharma Dental, Belgorod, Russia). Trioxident contains fine particles of calcium, silicon, aluminium oxides) with distilled water. The following component ratio was used: Trioxident 0.1 g + distilled water 0.25 mL. As was shown in [50], the creation of an alkaline environment (pH > 11) activates the formation of the functional molecular bonds calcium hydroxyapatite–amino acid across the surface of the cavity formed in the dental hard tissue, neutralizes the action of orthophosphoric acid, and reduces the formation of weak phosphate phases.

#### 2.2.5. Modified Adhesive

We added a powdered nanocrystalline carbonate-substituted hydroxyapatite HAp (1 mL of adhesive—0.01 g nano-c-HAp) to the original BisGMA-based liquid adhesive. Modification of the adhesive with HAp helps to increase the degree of polymerization, eliminate stresses that can be formed during polymerization, and contributes to the hardness of the layers [51,52]. HAp and adhesive were mixed using a QSonica 55 W ultrasonic homogenizer.

#### 2.2.6. Nanoscale Carbonate-Substituted Calcium Hydroxyapatite (HAp)

Powdered HAp was obtained using our technology from bird egg shells by liquid-phase synthesis [53]. The morphological organization of the synthesized HAp is similar to that of human tooth enamel apatite, as it is formed by nanocrystals with an average size of 20 × 20 × 50 nm [54]. This characteristic is a very important feature in the formation of a hybrid biomimetic interface capable of replenishing and integrating with natural apatite [55,56].

### 2.3. Microscopy

The enamel-dental composite interfaces were examined with a scanning electron microscope (JEOL, Tokyo, Japan) operating at 20 kV.

### 2.4. Synchrotron FTIR Microspectroscopy

The synchrotron FTIR experiment was performed on Infrared Microspectroscopy (IRM) beamline at ANSTO—Australian synchrotron (Victoria, Australia), using a Bruker Vertex 80v spectrometer coupled with a Hyperion 3000 FTIR microscope and a liquid nitrogen-cooled narrow-band mercury cadmium telluride (MCT) detector (Bruker Optik GmbH, Ettlingen, Germany). All the synchrotron FTIR spectra were recorded within a spectral range of 2000–850 cm^−1^ using 4-cm^−1^ spectral resolution. Blackman-Harris 3-Term apodization, Mertz phase correction, and zero-filling factor of 2 were set as default acquisition parameters using the OPUS 8.0 software suite (Bruker Optik GmbH, Ettlingen, Germany).

To avoid scattering artifacts commonly presented in reflectance spectra, the tooth slices were subsequently analyzed and imaged in macro ATR-FTIR mapping mode, using an in-house developed macro ATR-FTIR device equipped with a 250-μm-diameter facet germanium (Ge) ATR crystal (*n*_Ge_ = 4.0), and a 20× IR objective (NA = 0.60; Bruker Optik GmbH, Ettlingen, Germany) [44,57].

The unique combination of the high refractive index property of the Ge ATR crystal and the high numerical aperture (NA) objective used in this device, when coupled to the synchrotron-IR beam, allows surface characterization of the teeth slices to be performed without scattering artifacts and at higher spatial resolutions than those achievable in transmission and reflectance modes.

Prior to the macro ATR-FTIR measurement, the plane-parallel tooth segments (slices) were mounted on a flat polymer substrate using an epoxy adhesive (Moment, HENKEL, Moscow, Russia) and then mounted on an aluminium disc using double-sided polyimide (Kapton tape. The aluminium disc was then placed on the sample stage of the macro ATR-FTIR unit. After that, the Ge ATR crystal was brought to the focus of the synchrotron-IR beam, and a background spectrum was recorded in air using 256 co-added scans. The top polished surface of the teeth sample was then brought into contact with the Ge ATR crystal, and a low-resolution overview synchrotron macro ATR-FTIR chemical map was initially acquired to determine the area and quality of the contact, at a 5-μm step interval, using 8 co-added scans. A subsequent synchrotron macro ATR-FTIR mapping measurement was performed on specific areas of interest found in the prior overview map where a good contact with the Ge ATR crystal was achieved, using a smaller step interval of 1 μm and 32 co-added scans.

### 2.5. Multivariate Statistical Analysis

Multivariate data analysis to extract synthetic information when processing an array of statistical variables was performed using Hierarchical Cluster Analysis (HCA) and Principal Component Analysis (PCA).

Initially, the spectral information of the collected FTIR maps was classified using HCA. As a result, areas of spectral FTIR maps were identified based on the spectral response. The points on the map with minimal intra-cluster differences, i.e., belonging to one particular cluster, have a similar spectral response. At the same time, the maximum inter-cluster differences will be in areas with different spectral responses [58].

Initial processing of the initial spectral data during HCA was performed using the second derivative and vector normalization in the frequency range of the IR spectrum 2000–850 cm^−1^, since the main molecular vibrations of the materials under study are located in this region. The spectra were smoothed by 17 points.

The distance between the clusters was calculated based on the Euclidean measure. For clustering and construction of the heterogeneity dendrogram, the Ward method was used [59]. The number of clusters was determined based on the sample treatment data and the results of the heterogeneity dendrogram. Hierarchical Cluster Analysis (HCA) was performed using OPUS 8.0 software (Bruker Optik GmbH, Ettlingen, Germany).

The principal component analysis method, which is currently one of the preferred intelligent methods for processing and analyzing complex spectroscopic data [60,61], has been used to clearly identify or confirm correlations and similarities between a FTIR spectra of clusters. PCA reduces the dimensionality of the original spectral data set by computing a new set of variables, known as principal components, which are obtained in descending order of their contribution to the variance of the data. PCA was applied to a standard full range of FTIR spectra and performed using the first derivatives of the spectra. PCA was implemented in Matlab (R2013b, MathWorks, Boston, MA, USA). The preprocessing of the initial spectra, including background correction and noise reduction, was performed using a Savitzky–Golay filter.

## 3. Results and Discussion

**Figure 1:**
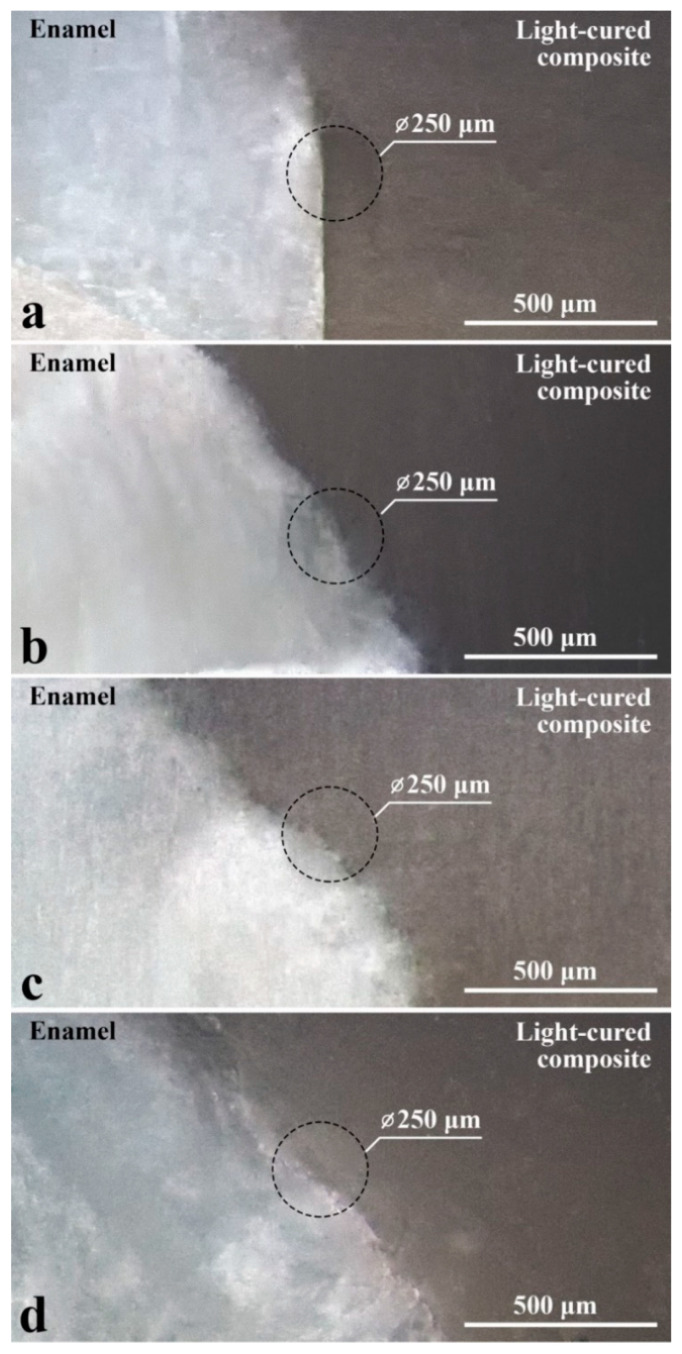
Optical images of the heterointerface sections of natural enamel-dental composite for the studied samples. (**a**) Sample S_I_; (**b**) Sample S_II_; (**c**) Sample S_III_; (**d**) Sample S_IV_.

The characteristic optical images obtained for the four types of prepared samples in the area of the natural enamel-dental composite interface are shown in Figure 1 with a magnification of 100×. For each type of sample (see Figure 1), two layers, namely enamel and dental composite, are clearly distinguishable.

**Figure 2:**
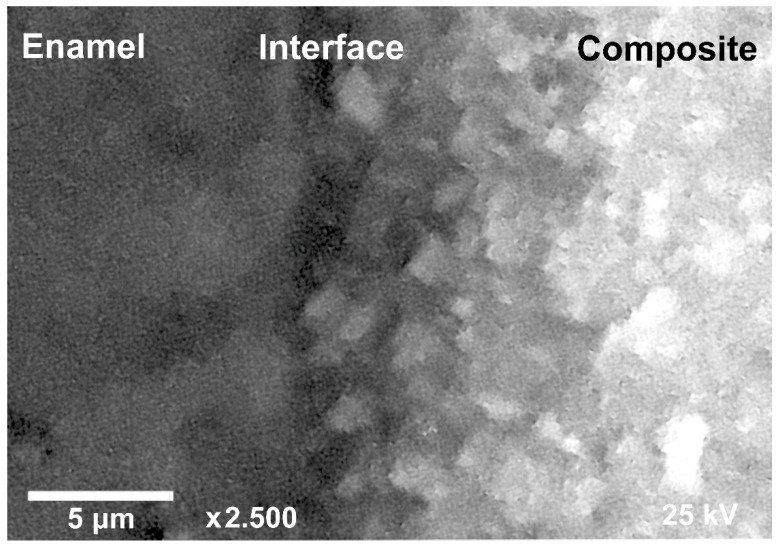
Typical SEM image of the enamel-dental composite heterointerface for the sample S_I_.

An SEM microphotograph of the typical section of enamel-dental composite interface for the sample S_I_ is presented in Figure 2. The analysis shows that irrespective of the pretreatment technique of a sample, formation of the clearly expressed hybrid layer at the interface is observed. It should be noted that such information can be obtained only from the chemical imaging, since both optical [62] and electron microscopic images [63] do not allow for the assessment of the occurrence of a chemical interaction in the interface region.

Using the FTIR optical system of the Hyperion 3000 microscope, we selected, for further analysis, those portions of the interface within which no mechanical defects from polishing or any other structural artifacts were visually observed.

The analysis of the chemical composition as well as the molecular features of the samples in the enamel-biocomposite interface was performed using the FTIR technique. In contrast to a number of other methods of molecular composition analysis (e.g., Raman spectroscopy), when using the FTIR technique, samples of a biological nature are subjected to weak external influences when under study. Therefore, the information obtained refers to a system that has not undergone changes as a result of these interactions, and changes in the interface region caused by molecular transformations can be easily identified.

**Figure 3:**
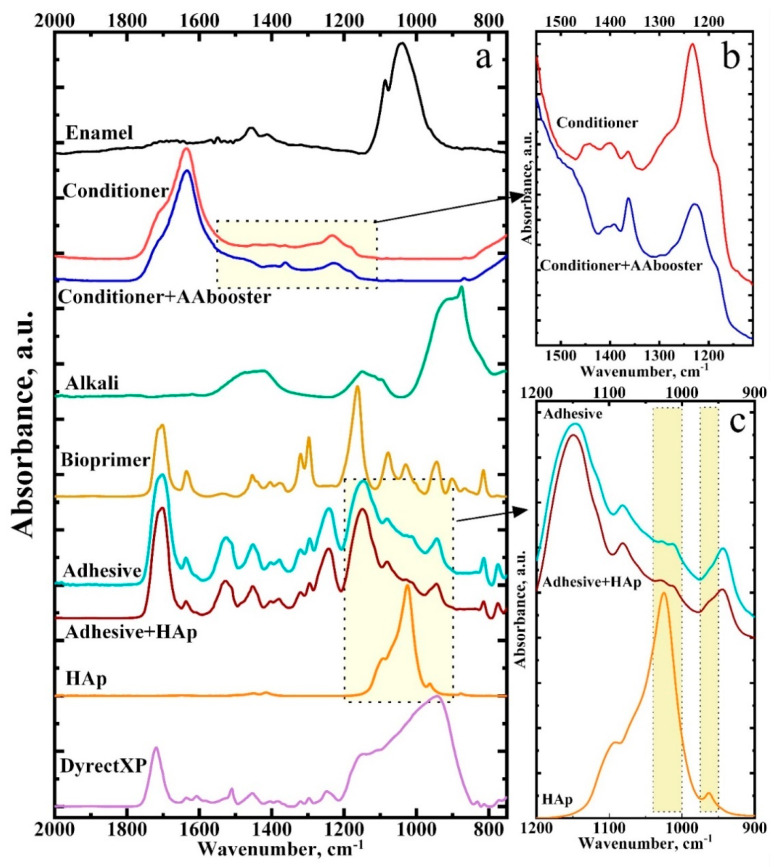
Typical FTIR spectra of the specimens studied in this work. (**a**)—spectra for the healthy enamel, DyractXP dental compomer material, the conditioner including the amino acid booster, bioprimer, calcium alkali, original and modified BisGMA-based adhesive, and the nanocrystalline carbonate-substituted calcium hydroxyapatite; (**b**)—FTIR spectra in the region of the most pronounced transformations of the vibrational modes for conditioner and conditioner including the amino acid booster; (**c**)—FTIR spectra in the region of the most pronounced transformations of the vibrational modes for HAp, BisGMA-based adhesive and modified BisGMA-based adhesive.

Figure 3 shows the representative FTIR spectra of substances (chemical components) present in the interface area of all samples, as well as the materials we used to create a hybrid interface. Thus, Figure 3 shows the absorption spectra in the infrared fingerprints region for the healthy enamel, DyractXP dental compomer material, the conditioner including the amino acid booster, bioprimer, calcium alkali, original and modified BisGMA-based adhesive, and the nanocrystalline carbonate-substituted calcium hydroxyapatite (HAp). For convenient analysis of the changes in the molecular spectrum of the conditioner and adhesive after their modification, Figure 3b,c presents the IR spectra in the region of the most pronounced transformations of the vibrational modes.

It should be noted that the relative intensities of the main oscillations of the chemical substances mentioned depend on the sample preparation or the individual (as in the case of the spectrum of healthy enamel) from whom the sample was taken. Therefore, the spectra presented in Figure 3 are averaged, typical for the material, and consistent with previously published works [25,64,65,66,67,68].

Table 2 lists the frequencies of the characteristic oscillations and their belonging to functional groups of the substances from the interface area, such as enamel, adhesive, and dental composite, as well as the substances used for treatment.

The simultaneous use of optical microscopy and FTIR spectroscopy allowed us to obtain molecular information from the interface regions with high spatial (~500 nm) and spectroscopic resolution. For this purpose, selected regions (20 μ × 50 μ or 40 × 100 pixels) were analyzed using the synchrotron micro-ATR FTIR mapping technique. Every FTIR-spectrum was collected with a beam defining aperture providing a nominal measurement area of 3.13 μm diameter per pixel, at 500 nm step intervals and without scattering artifacts.

To obtain the chemical information (molecular differentiation), chemical images were processed based on the variation of spectral line intensities reflecting the distribution of the characteristic molecular groups in the interface region. For this purpose, four important absorption bands in FTIR spectra attributed to the molecular groups of materials that are present in the interface region were selected [62].

The first spectral line, with a maximum at 1726–1728 cm^−1^, correlates with vibrations of the ester group (-COOCH_3_) of the organic matrix included in the BisGMA adhesive and also present in the DyractXP dental material [84].

The second line in the region of 1700–1620 cm^−1^ corresponds to the vibrations of the Amid I group, characteristic of the organic component of enamel (proteins) [64,69], which fills the interprism space. In addition, the oscillations of the N-H, C=O, and COO- molecular groups of the amino acids that make up the bioprimer and booster used in the creation of the interface are located in the region of 1700–1620 cm^−1^ [84,85,86].

The third peak of 1430–1370 cm^−1^ is associated with vibrations of the CH and COO- groups [22] belonging to the amino acid booster, conditioner, and bioprimer.

The fourth spectral line, 1110–960 cm^−1^, is the sum of overlapping bands active in the FTIR spectrum of enamel apatite, which are associated with phosphate ions—PO_4_^3−^ [37,64,68]. The most intense here is the υ_3_ PO_4_^3−^ Antisymmetric stretching mode, localized around 1038 cm^−1^, which is characteristic of non-stoichiometric calcium hydroxyapatite. According to [68], the shift of the main maximum of the υ_3_ PO_4_^3−^ band and the appearance of a fine structure near it indicate a change in the chemical composition and the formation of various calcium phosphates. Therefore, a broad spectral region, including possible variations in the υ_3_ υ_3_ PO_4_^3−^ position, was chosen to analyze the interface. The region chosen for mapping (about 1110 cm^−1^) contains a band characteristic of apatites containing HPO_4_^2−^ ion, as well as a line about 1090 cm^−1^ [70], characteristic of the PO_4_^3−^ phosphate ion in nanocrystalline carbonate-substituted hydroxyapatite [71]. Moreover, the Si-O bond oscillations of silicon dioxide used as a filler for the composite dental material are also in the same region [86].

**Figure 4:**
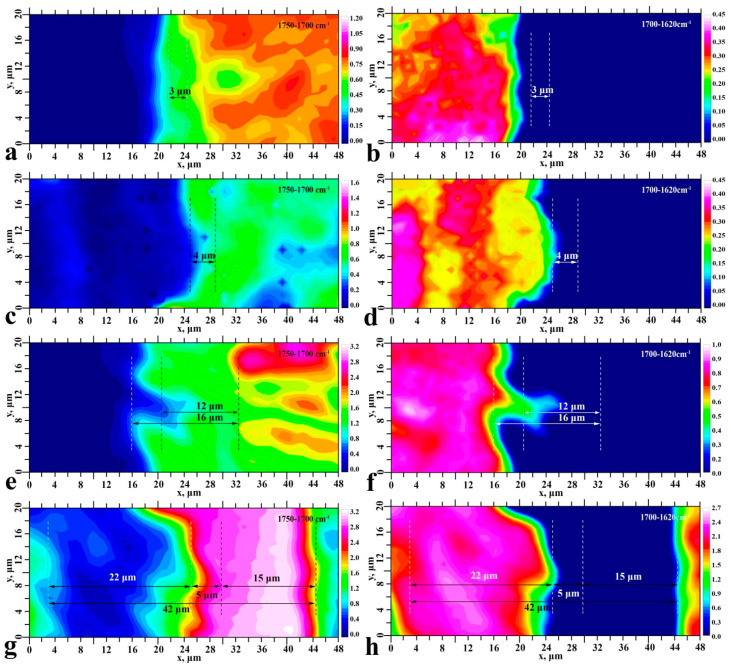
Chemical images of typical surface areas and reflecting the spatial distribution in the interface region (see Figure 1) of -COOCH_3_ (**a**,**c**,**e**,**g**) ester group vibrations and Amid I vibrations (**b**,**d**,**f**,**h**). Sample S_I_—(**a**,**b**); sample S_II_—(**c**,**d**); sample S_III_—(**e**,**f**); sample S_IV_—(**g**,**h**).

**Figure 5:**
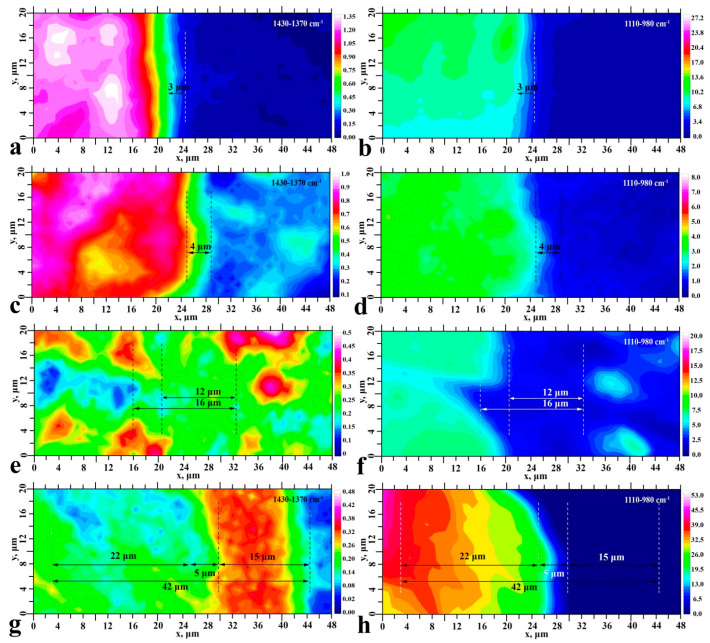
Chemical images of typical surface areas reflecting the spatial distribution in the interface region (see Figure 1) of the CH and COO- group vibrations (**a**,**c**,**e**,**g**) and phosphate vibrations (**b**,**d**,**f**,**h**). Sample S_I_—(**a**,**b**); sample S_II_—(**c**,**d**); sample S_III_—(**e**,**f**); sample S_IV_—(**g**,**h**).

Finding the distribution of the integrated area value under the selected absorption lines in the mapping area allowed us to create chemical images of the interface for each type of investigated samples. The resulting maps are shown in Figure 4 and Figure 5. The chemical images constructed give us information on the spatial distribution of mineral (phosphates) and organic (enamel proteins, amino booster and bioprimer components) components in the interface area, as well as the adhesive and dental material for all sample types.

Preliminary analysis of the chemical images shows that each type of formed enamel-dental composite interface represents a transition layer. The width of this layer may implicitly indicate the occurrence of interaction between the materials used to create the interface. The FTIR maps of samples S_I_–S_III_ clearly show a non-homogeneous distribution of the ether (-COOCH_3_) band intensity, and for sample S_IV_, a characteristic zoning (layer-by-layer) distribution of the adhesive in the interface zone is observed. One can notice from Figure 4 and Figure 5 that the width of the transition layer depends on the type of the created interface.

As has been repeatedly noted previously, the analysis of only chemical images of the interface showing the distribution of the dental material, and the organic and mineral component of the enamel, often does not allow us to reveal the molecular features of the created interface, or reveal the mechanisms of the integration processes between the natural dental hard tissue and the biocomposite material. This is due to the inability of the used approach to identify and take into account insignificant spectral changes associated with the nucleation of the chemical interaction at the interface. Frequent overlapping of spectral lines does not allow one to confidently analyze transition layers of close and gradient composition in the region of integration. In our case, the overlap of the bands in FTIR spectra can be observed in the 1100–960 cm^−1^ region, where several vibrations associated with the phosphate groups of the PO_4_^3−^ mineral component of enamel as well as the Si-O vibrations of silicon dioxide included in the dental material [83,84], or in the 1700–1200 cm^−1^ range, which is the fingerprint for the organic structure. However, these inconveniences can be overcome by using multidimensional methods for analyzing a large set of spectroscopic data, allowing for the efficient processing and systematization of an array of spectra of multicomponent materials collected as 2D FTIR maps [87,88,89], and detecting and confirming the relationships and similarities between FTIR spectra in the set. Such methods include hierarchical cluster analysis (HCA) and the principal components method (PCA). Using multivariate statistical methods to analyze an array of spectra of multicomponent systems is useful for identifying information that might have been missed in a simpler analysis. This is especially important for biological samples, which by their nature contain heterogeneous molecular compounds [90].

**Figure 6:**
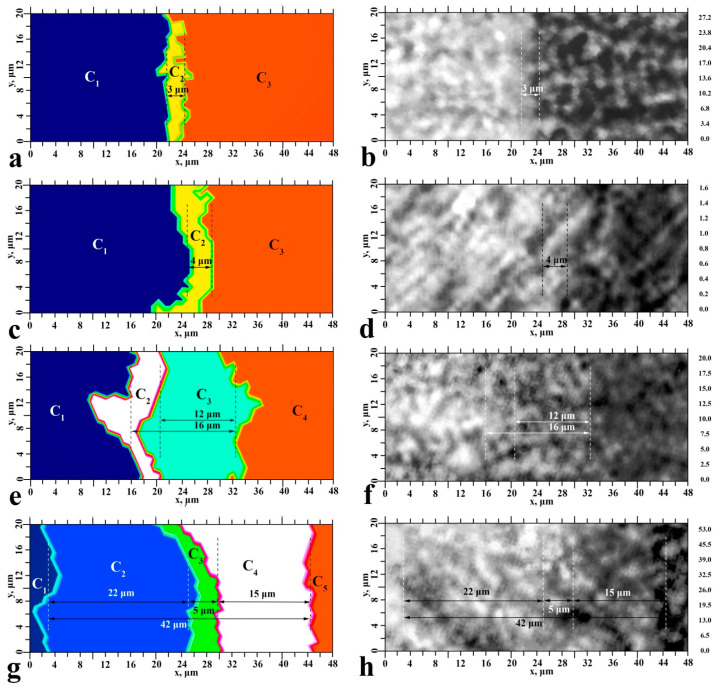
Results of the cluster analysis for the studied samples (**left**) and optical images of ×100 sections in the interface region (**right**). Sample S_I_—(**a**,**b**); sample S_II_—(**c**,**d**); sample S_III_—(**e**,**f**); sample S_IV_—(**g**,**h**).

The results of the cluster analysis (see Figure 6, left), as well as the results of chemical mapping (Figure 4 and Figure 5), are presented using colour coding. All points that have a similar spectral response within a certain region (cluster) are indicated by the same colour. Thus, the figures show groups of areas (clusters) with a different spectral response (see Figure 6, left). Note that the number of clusters for each type of interface was determined taking into account the following criteria: sample data, information from the heterogeneity diagram, and a low signal-to-noise ratio. Figure 6 (right) also shows optical images of the enamel-biocomposite interface section. It should be noted that the change in contrast in the optical images (see Figure 6, right) does not give a clear indication of the boundaries of specific chemical zones (clusters). However, the HCA results clearly show that the number of clusters in the hybrid layer, as well as their sizes, depend on the type of sample preparation.

Thus, in the hybrid interface zone (between the enamel area and the dental composite), samples S_I_ and S_II_ have only one cluster with a width of 3–4 μ. As the number of technological steps in the formation of the interface increases, sample S_III_ forms two clusters, and sample S_IV_ has three clusters of different thickness. The total width of the formed hybrid layer according to the HCA results correlates with the value determined from the chemical imaging data.

The chemical composition of each cluster was established based on the analysis of averaged cluster spectra. The FTIR spectrum of each cluster was obtained by averaging at least 50 individual spectra from the corresponding zones of similar samples. Before analysis, the averaged cluster spectra were processed (smoothing, baseline correction, vector normalization) using standard Bruker OPUS 8.0 software procedures. As a result, the values of the clusters present were identified for each interface type, such as enamel, hybrid layer, adhesive, and dental composite.

**Figure 7:**
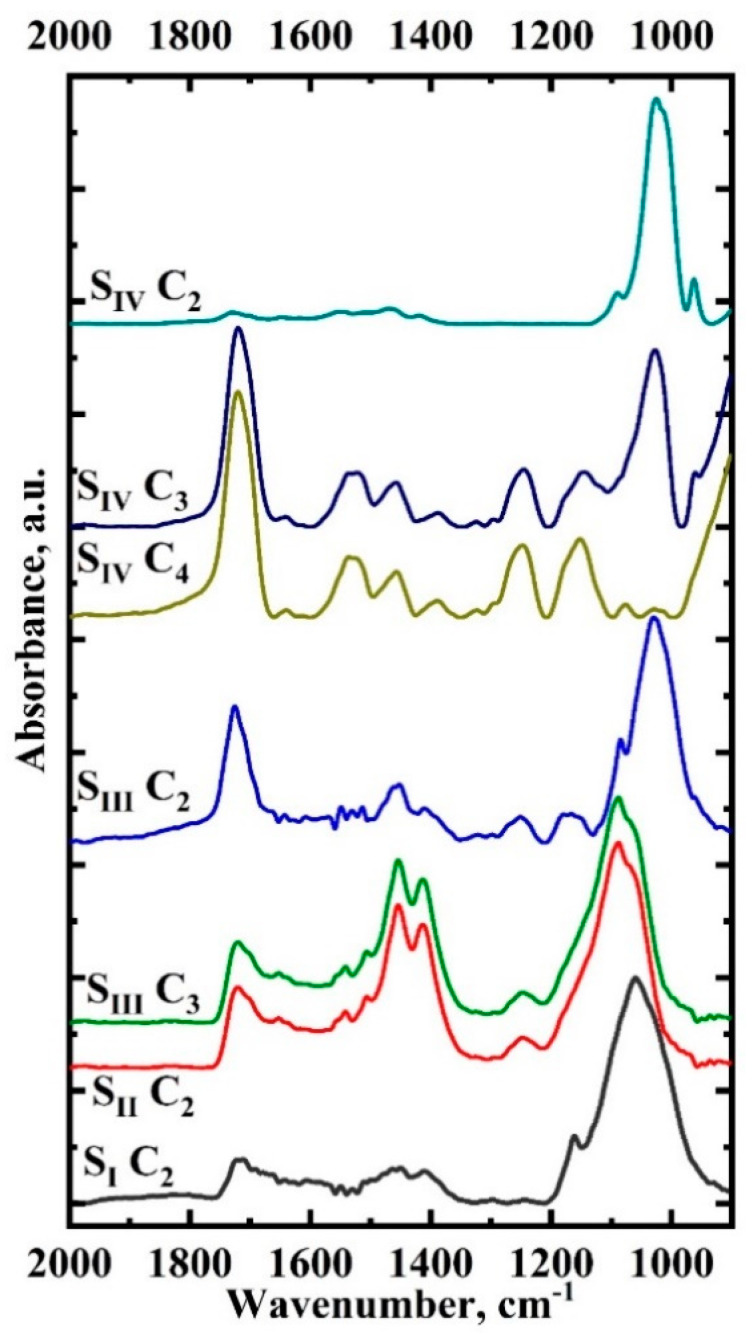
Averaged spectra of clusters belonging to the transition/hybrid layer for each type of samples studied.

Figure 7 shows the averaged spectra of the clusters belonging to the transition/hybrid layer for each type of sample studied. We do not present the spectra of the clusters associated with the enamel and dental composite due to their uniformity but consider only the spectra of the clusters associated with the hybrid layer. In addition, we should note the fact that the area we are considering (Figure 4 and Figure 5) is 20 μ × 48 μ, which is comparable to the size of enamel prisms ~ 5 μ [2]. Chemical and molecular analysis of the array of enamel prisms shows that there are local imperfections and a differentiation of chemical and phase composition already within neighboring prisms and the area between them [1]. Thus, the averaging of spectra within a single region (cluster) to smooth the variation of the chemical composition is important when the analysis area is reduced.

Analysis of the obtained results shows (see Figure 7) that there are noticeable variations (position and relative intensity of absorption bands) in FTIR spectra of hybrid interface clusters depending on the type of sample (interface). These variations are due to the structural and chemical differentiation of the layers of the interface being formed, as well as to their interaction. Analyzing simultaneously the HCA data (Figure 6 and Figure 7) it can be observed that the adhesive components are present in all clusters of the studied samples, which is detected by the presence in the spectra in the region of the 1720 cm^−1^ band correlated with the vibrations of the ester group (-COOCH_3_). At the same time, in the C_2_ clusters, which border the enamel zone, the relative intensity of this vibration changes. Analysis of the spectra shows that for the S_I_ and S_II_ samples, the adhesive is an intermediate layer between the enamel and dental composite (Figure 3 and Figure 7). The components of the adhesive penetrated minimally into the enamel microporosity array and also diffused into the boundary region of the biocomposite. In sample S_III_, the adhesive was present in clusters C_2_ and C_3_ in close concentrations. In the S_IV_ sample, on the other hand, a concentration (penetration) gradient of the modified adhesive in the direction from the composite to the enamel was observed. In addition, components of the bioprimer and amino acid booster are detected in intermediate clusters C_3_ and C_4_ of the S_IV_ sample.

In addition to the HCA results, a multivariate spectral image (cluster) analysis using PCA was applied. The analysis was performed in the same spectral range of 900 to 2000 cm^−1^ to automatically distinguish the interface zone components at subnanostructural levels. This type of analysis is performed to compare the effect of the treatment type on interface formation by interpreting the similarities and differences between clusters belonging to the transition layer of samples of different types by analyzing the PCA scores and loadings. Here, PCA loadings are the combinations of FTIR spectra of the components of the hybrid sample layer, which allow for differentiating (determining the largest difference) the samples into classes.

**Figure 8:**
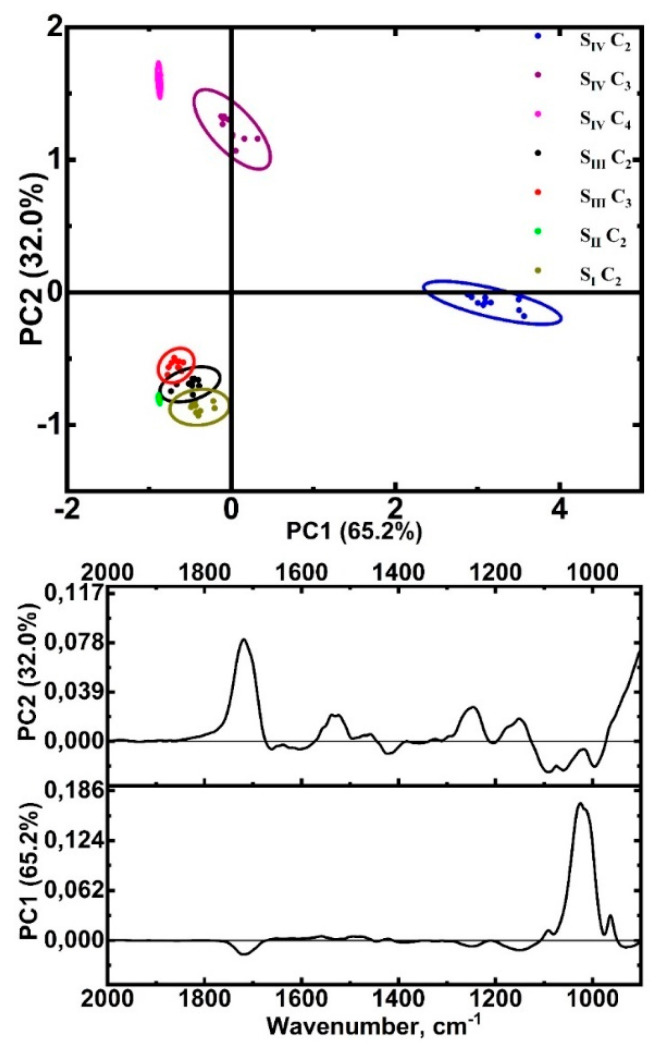
Results of the PCA analysis of interface zone components (clusters) for all samples studied. Above is the PCA scores plot (PC1 vs. PC2), and below are the PC loading plots for the PC1 and PC2.

The clusters formed at HCA, visualized in the chemical interface images, and correlated with the hybrid interface layer region, can be clearly observed in the PCA plots (Figure 8, top). Each zone cluster in the interface region is represented by an ellipse for a particular sample type. Figure 8 (bottom) also shows the load plots for the main components as a function of wave number. The clusters formed at HCA, visualized in the chemical interface images, and correlated with the hybrid interface layer region, can be clearly observed in the PCA plots (Figure 8, top). Each zone cluster in the interface region is represented by an ellipse for a particular sample type.

It is well seen that the PCA score plots demonstrate a clear separation between the interface zones for the S_IV_ sample. At the same time, the ellipses corresponding to the clusters of samples S_I_, S_II_, and S_III_ are clustered and partially overlap, indicating the closeness of the spectra (molecular properties) of these interface areas. At the same time, most of the differences between the hybrid layer clusters in different samples can be explained by the PC1 (65.2%) and PC2 (32.0%) components.

The load plots are shown in Figure 8 for each sample type and visualize the set of peaks that contribute most to the observed differences between the interface zones. Table 3 displays the wave numbers of the characteristic maxima and minima that are present in the load plots of the PCA samples and indicates the likely functional groups attributed based on a literature search [25,37,64,65,66,67,68,83,84].

According to the analysis of the PC1 and PC2 principal component loadings plots (see Figure 8), the peaks associated with phosphates, adhesive components, and the amino acid booster contributed most to the differentiation of clusters of different samples (the main contribution to the observed differences).

Analyzing the results obtained, it should be noted that in the spectra of the hybrid layer clusters of S_I_, S_II_, and S_III_ phosphate samples, the maximum is shifted to the region of 1050–1060 cm^−1^, while for natural tooth enamel apatite, it is localized around 1038 cm^−1^. This fact is related to the pretreatment of enamel, namely acid etching followed by a single treatment with calcium alkali. Exposure to acid breaks the Ca-O bonds and, consequently, reduces the P-O bond length due to a redistribution of the electron concentration near the bridging oxygen [91]. As a result, weak phosphates, bruchite, and TCF, whose characteristic bands lie in the region of 1060–1070 cm^−1^, are formed [37,68]. It should be noted that no phosphate band shift is observed for the S_IV_ sample. The repeated process of treatment with calcium alkali and the introduction of organic acids into the interface zone minimized the formation of stable brushite-like forms and octacalcium phosphate, whose formation is possible only on the surface of apatite nanocrystals. Subsequent processes of the redeposition of HAp nanocrystals with the formation of a non-apatite environment led to the appearance of a maximum in the spectra of the S_IV_ sample clusters in the infrared spectrum near 1107 cm^−1^. A similar result was described in [92], and this oscillation near 1090–1110 cm^−1^ can be attributed to the vibration of the secondary Ca-O-P phase [91,93]. Thus, the use of amino acids and an alkaline solution for pretreatment and biointerface formation results not only in an excess of calcium in the hybrid transition layer but also in the creation of the conditions for calcium binding to phosphate complexes (HPO_4_ and PO_4_) [94]. Moreover, the features of the FTIR spectrum of the C_2_ cluster in the S_IV_ sample give a reason to state that this interface layer is a reduced enamel zone. The narrowing of the phosphate peak as well as the absence of the carbonate group in the C_2_ spectrum of the S_IV_ sample indicates the formation of highly crystalline apatite in this rather wide region of the interface.

The results of work on the analysis of the surface layers of nanocrystalline biogenic and synthetic apatite [94] have already demonstrated differences in the local atomic and molecular environment of calcium atoms, which may affect the formation of the HAp–organic matrix bond [22]. As studies on the surface of biogenic apatite nanocrystals have shown, the environment of calcium atoms corresponds to the Ca-ON environment [94,95]. Therefore, in the S_IV_ interface formation process under consideration, the repeated calcium alkali treatment process can contribute to the formation of conditions when the pretreated enamel surface after etching has bonds characteristic of the enamel apatite complex before treatment and contribute to the formation of bonds with amino acids in the composition of the booster used.

In addition, the results of chemical imaging, HCA and PCA analyses show that the hybrid interface region (clusters C_2_ and C_3_) of S_I_–S_III_ samples is formed by the non-homogeneous distribution of the adhesive as well as the materials used to create the hybrid layer. At the same time, the effect of gradient and the layer-by-layer distribution of the materials used in the clusters is characteristic of the S_IV_ sample. Due to the diffusion of the amino acid booster conditioner component and the modified HAp adhesive, a structure is formed in the hybrid interface region, which should stabilize the reconstituted crystalline enamel layer. At the same time, HAp nanocrystals contribute to the cohesive reinforcement of the adhesive layer, as they are evenly distributed throughout it. This leads to increased adhesion and improved conversion of the adhesive resin in the hybrid layer, as well as better micromechanical bonding [52].

To summarize the work performed, it should be noted that the use of nanotechnology for the fabrication and functionalization of biomimetic dental restorative composites is a very promising area. Based on the information obtained in our work, not only new biomaterials that mimic the properties of natural tissue but also innovative biomimetic strategies that provide controlled stable mineralization and biomechanical properties of the hybrid layer are needed to form an ideal bonding. This would ultimately lead to minimal invasion, improved restoration quality, and a reduced incidence of secondary caries.

It should be noted that in spite of the formation of the tight bond at the interface of the enamel-biocomposite, a rather important task is to make it stable in time, in the acidic environment with pH <5, as well as under cyclic impact of various temperature levels. These studies will be the aim of our future work.

## 4. Conclusions

Using a biomimetic strategy and bioinspired materials, our work proposes a new technological approach to create a hybrid transitional layer between the enamel and dental biocomposite. For this purpose, an amino acid booster conditioner based on a set of polar amino acids (lysine, arginine, hyaluronic acid), calcium alkali, and a modified adhesive based on BisGMA and nanocrystalline carbonate-substituted hydroxyapatite HAp are used at the stage of dental enamel restoration.

The molecular properties of the hybrid interface formed using the proposed strategy were understood using methods of multivariate statistical analysis of spectral information collected, applying the technique of synchrotron infrared microspectroscopy.

The results obtained indicate:

- the use of amino acids and an alkaline solution for pretreatment and biointerface formation results not only in an excess of calcium in the hybrid transition layer but also in the creation of conditions for calcium binding to phosphate complexes (HPO_4_ and PO_4_);
- the repeated calcium alkali treatment process can contribute to the formation of conditions where the pretreated enamel surface, after etching, has bonds characteristic of the enamel apatite complex before treatment, and contributes to the formation of bonds with amino acids in the composition of the booster used;
- during diffusion of the amino acid booster conditioner component and the modified HAp adhesive, a structure is formed in the hybrid interface region, which should stabilize the reconstituted crystalline enamel layer.

The developed technology can become the basis for an individualized, personalized approach to dental enamel restoration.

## Figures and Tables

**Table 1:** Technological operations when creating interfaces.

Samples	Alkali	Amino Acids Booster in Dental Conditioner	Conditioner Treatment	Bioprimer	BisGMA Adhesive	HAp in BisGMA Adhesive	DyractXP
S_I_	−	−	+	+	+	−	+
S_II_	+	−	+	+	+	−	+
S_III_	+	+	+	+	−	+	+
S_IV_	++++	+	++	+	−	+	+

**Table 2:** Main vibrations in the infrared spectra and their belonging to the functional groups of the materials or substances involved in the study.

Substance/ Material	Spectral Area, cm^−1^	Functional (Molecular) Group	References
Enamel	1620–1700	Amide I C=O stretching	[64,69]
	1550	Amide II C─N stretching and N─H deformation modes, CNH	[64,69]
	1535	OH^−^ substituted by CO_3_^2−^ (type A)	[70,71]
	1465 1417	PO^3−^ substituted by CO_3_^2−^ (type B)	[70,71]
	1130	HPO_4_^2−^	[68,72]
	1030	HPO_4_^2−^	[64,70,73]
	960	υ_1_ PO_4_^3−^ Symmetric stretching	[64]
HAp	1460	PO_4_^3−^ substituted by CO_3_^2−^ (type B)	[25,70]
	1415	PO_4_^3−^ substituted by CO_3_^2−^ (type B)	[25,70]
	1092	υ_3_ PO_4_^3−^ Antisymmetric stretching	[25]
	1040	υ_3_ PO_4_^3−^ Antisymmetric stretching	[37]
	963	υ_1_ PO_4_^3−^ Symmetric stretching	[25]
Conditioner + Amino acids booster	1718	C=O carbonyl group of AA	[74]
	1635	vas, COO- vas(CN_3_H^+5^)	[50]
	1460–1445	CH_2_/CH_3_	[50]
	1362	N-Cα-Hα, Cβ-Cα-Hα	[50]
	1226	NH_3_^+^	[50,75]
	1185	ρ, NH_3_^+^	[50]
Bioprimer	1703	C=O stretching	[76]
	1635	vas(CN_3_H^+5^) protein amino acid, arginine	[77,78]
	1454	–CH_2_	[79]
	1320–1298	[v(C-O)] stretch doublet δ(CH)	[76]
	1164	C-O-C, δ(CH)	[76]
	1078	ν(CO)	[79]
	1026	ν(CC)	[79]
BisGMA Adhesive	1721	C=O carbonyl	[80]
	1636	C=C Aliphatic C=C methacrylate groups	[80]
	1609	phenyl C=C	[80,81]
	1513	Aromatic C=C	[80]
	1452	CH_2_ CH_3_	[80,81]
	1402	=CH_2_ deformation	[46]
	1320–1290	[v(C-O)] stretch dublet	[46]
	1242	Aromatic C–O	[46]
DyractXP commercial material	1700–1740	Ester groups -COOCH_3_ attached to the methacrylate	[82]
	1636	C=C stretching vibration of the methacrylate group	[82,83]
	1608	C=C in an aromatic ring	[82,83]
	1511	N–H deformation stretching of urethane dimethacrylate (UDMA)	[82,83]
	1454	C–H in constituent monomers	[83]
	1297	symmetric stretching of -O in monomers, Si–O stretching	[83]
	1233	C–O–C stretching	[82]
	1150	C–O–C stretching	[82]
	1040–1060	Si–O from SiO_2_ -containing fillers	[83,84]

**Table 3:** Results of PC loading plots for PC1 and PC2: wave numbers of the most prominent peaks and troughs in the PCA loading plots and their correlation with functional groups based on a literature search (see Table 2).

	Wavenumber, cm^−1^											
PC1	961		1014	1025								1718
PC2		995			1059	1092	1149	1248	1448	1522	1538	1720
Assignment	υ_1_ PO_4_^3−^ enamel nano-c-HAp	DCPD and β-TCP	υ_3_ PO_4_^3−^	the stoichiometric apatites containing HPO4 ^2−^ ion	DCPD and β-TCP	υ_3_ PO_4_^3−^ nano-c-HAp	HPO_4_^2−^ stoichiometric apatites	Aromatic C–O	PO^3^^−^ substituted by CO_3_^2−^ (type B) CH_2−_CH_3_	Aromatic C=C	δ^s^NH_3_^+^	BIS-GMA

## Data Availability

The data that support the findings of this study are available from the corresponding author upon reasonable request.
